# Supplementary figures and images for: Leveraging word embeddings to enhance co-occurrence networks: A statistical analysis
Source: PLoS One. 2025 Jul 11;20(7):e0327421. doi: 10.1371/journal.pone.0327421 (PMC12250493; doi:10.1371/journal.pone.0327421)

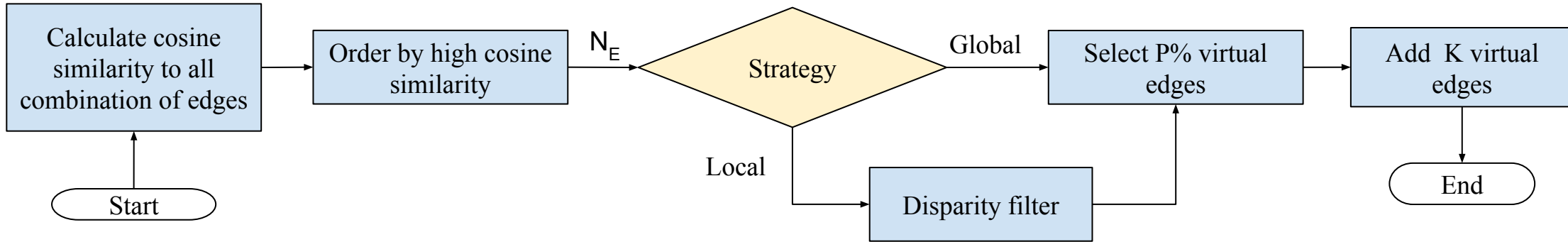

Supplement: S1 Fig — (PDF) [file pone.0327421.s001.pdf]

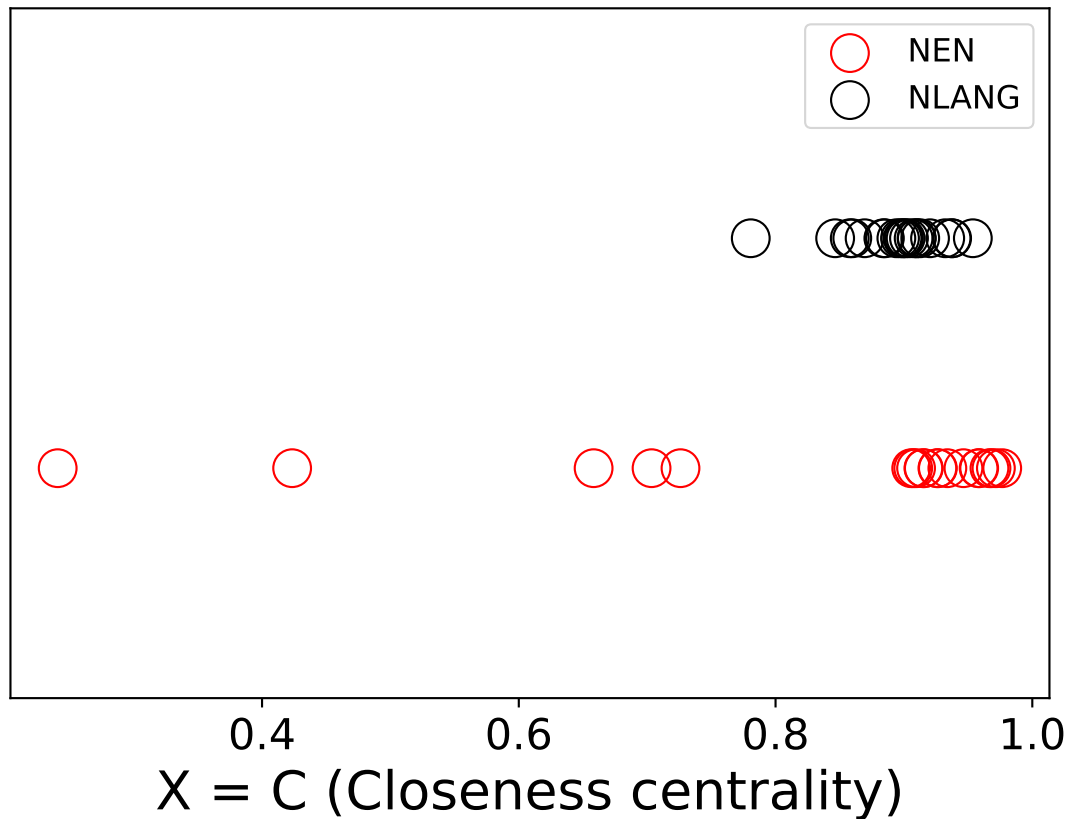

Supplement: S2 Fig — (PDF) [file pone.0327421.s002.pdf]
